# Strengthening Device for Improving Shear Performance of Anchor Cable in Rock Support

**DOI:** 10.3390/ma17010197

**Published:** 2023-12-29

**Authors:** Chao Feng, Shaowei Liu, Housheng Jia, Mengxiong Fu, Deyin He

**Affiliations:** 1 School of Energy Science and Engineering, Henan Polytechnic University, Jiaozuo 454000, China; xyfengchao0518@163.com (C.F.); jiahousheng@126.com (H.J.); fusupport@126.com (M.F.); hdyulf@126.com (D.H.); 2 School of Architecture and Civil Engineering, Anyang Institute of Technology, Anyang 455000, China; 3 Collaborative Innovation Center of Coal Work Safety and Clean-Efficiency Utilization, Jiaozuo 454000, China

**Keywords:** rock support, anchor cable, tensile–shear failure, double-shear test, numerical simulation

## Abstract

Many designs of anchor cables are currently in use for rock support in civil and mining operations. Because of the exposed surface and weak shear performance of the cable bolt’s free section (CBFS) in end-anchored structures, breaking failure frequently occurs. Numerical simulations and laboratory experiments were performed in this study to develop measures to improve CBFS resistance to shear failure. Analysis of shear characteristics of the CBFS showed that higher axial tension weakens the cable bolt’s shear resistance, and that shear damage on the cable surface and uneven distribution of shear stress aggravate CBFS tensile–shear failure. A high-strength steel pipe is proposed to protect the shear cable bolt, and the preliminary design of a CBFS-strengthening device (CFSD) is presented. Numerical simulation revealed that the CFSD effectively improved CBFS shear resistance and provided protection from harmful shear damage. The optimal performance of a Q-type (slotted steel pipe) CFSD was confirmed. The mechanism of improvement of the cable’s shear resistance to surrounding rock by employing the CFSD was analyzed. Double-shear tests were carried out on a bare cable bolt and a cable bolt with a Q-CFSD. The results revealed that the CFSD increased the peak shear force on the joint plane, cable peak axial force, and ultimate shear displacement by 31%, 18%, and 11%, respectively. The proposed device is effective in improving the shear performance of end-anchored cable bolts and enhancing surrounding rock stability.

## 1. Introduction

Anchor cables and bolts technology plays a very important role in rock support engineering in civil and mining operations [1,2,3,4]. The failure of the free section of anchor cables due to unstable rock shear action is a common form of failure in engineering practice, which severely restricts the development of anchor cable support technology [5,6,7,8,9]. Because the cable-bolt free section (CBFS) passes through the loose mass of shallow rock, it not only transmits an axial tensile load, but also bears a transverse shear action caused by rock mass sliding. The exposed surface and weak shear resistance of the CBFS exacerbate its shear failure. Therefore, to maintain surrounding rock stability, it is of great significance to protect the CBFS and improve its shear resistance.

In recent years, there has been increasing attention on the shear characteristics of cable bolts, and much experimental research has been carried out. Mirzaghorbanali et al. [10] investigated the influence of the prestress and surface profile on cable shear strength using an improved double-shear tester. Their results revealed that increasing prestress reduced the ultimate shear displacement and peak shear value of the anchor cable. Aziz et al. [11,12] developed and improved equipment suitable for cable-bolt shear testing and conducted in-depth research on the shear behavior of different types of anchor cables. In their most recent research, they conducted double-shear experiments on anchor cables at different anchoring angles and found that an increased angle of shear contributes to increased stiffness of the cable in shear [13]. Li et al. [14,15] systematically investigated the shear mechanical behavior of a grouting anchor cable and its contribution to joint-plane shear strength using theoretical analysis and experimental comparison. Craig and Aziz [16] studied the shear performance of a 28 mm TG hollow cable bolt and found that cable-bolt failure was caused by the cable axial load near the structural plane reaching the tensile strength. Rasekh et al. [17] investigated the influence of the ultimate tensile strength, anchorage length, number of steel strands, and surface profile of cable bolts on the shear strength of a grouting anchor cable using a double-shear test. Yang et al. [18] experimentally investigated the shear characteristics of an end-anchored CBFS and reported that shear failure of the CBFS was caused by the combination of transverse shear and axial tensile loading. Wang et al. [19] conducted large-scale single-shear tests on prestressed anchor cables and analyzed their failure characteristics. The abovementioned studies elucidated the cable-bolt shear mechanical characteristics and shear fracture mechanism, but most considered a full-length anchor cable, for which the mechanism is quite different from that of an unanchored CBFS.

High-strength cable bolts add difficulty to laboratory shear tests. Many scholars have carried out more economic and effective numerical simulation analyses of the shear characteristics of cable bolts. Aziz et al. [13] found that UDEC and 3DEC software achieved good numerical simulation of shear mechanical characteristics of anchor cables at different angles and prestress conditions. Mirzaghorbanali et al. [10] used FLAC 2D to numerically simulate the peak load strength of prestressed grouted anchor cables, and the simulation outputs matched well with the experimental results. Li et al. [20] simulated the shear behavior of cable bolts using calibrated pile structural elements in FLAC 3D and obtained reasonable anchor lengths along the anchor interface. Sun et al. [21] proposed mechanical criteria and constitutive models for the yielding and fracture of rock bolts under tensile–shear coupled loads and achieved secondary development of the FLAC 3D platform. Saadat et al. [22] proposed a new cohesive contact model in distinct element codes (PFC 2D) to investigate the mechanical performance of fully grouted rock bolts. Tahmasebinia et al. [23,24] and Wang et al. [25] established numerical models of shear tests on cable bolts using the commercial ABAQUS finite-element package to study the dynamic and static shear mechanical characteristics of cable bolts. Numerical simulation is an indispensable method for studying the shear mechanical characteristics of cable bolts and can effectively compensate for shortcomings of laboratory experimental research in monitoring the entire process of microscopic failure evolution. However, the above research focused on simulating full-length anchoring cables; there are few reports on the numerical simulation of shear mechanical behavior of the end-anchored cable free section. Moreover, most scholars have simplified the cable bolt as a circular bar, which ignores the influence of the bundled structure on the transverse shear effect.

With the aim of achieving low shear resistance of the cable bolt, one of the main improvements is the use of full-length anchorage or grouting anchor cable technology [26]. Although a hardened anchoring agent wraps the cable bolt to provide greater lateral resistance, this technology extends the construction process, while anchorage interfacial adhesion limits the axial tensile load transmission of the CBFS [27]. Shan et al. [28,29,30] proposed a pipe–cable composite structure (anchor cable with a C-shaped pipe) and reported that this structure could effectively improve the overall shear capacity of the structural plane. Kang et al. [31] reported that, by increasing the cable diameter and changing the cable structure, a strong and high-elongation cable bolt can effectively control the expansion deformation of surrounding rock in the anchorage zone. Li et al. [32] proposed a new supporting structure with a high-strength cable bundle formed by multiple cable bolts to improve the tensile and shear strengths of the supporting components. He et al. [33] investigated the shear properties of a micro-negative Poisson’s ratio steel bolt and showed that this had higher tensile and shear strength than an ordinary steel bolt and strong energy-absorption properties. However, with an unclear understanding of the actual shear failure mechanism of the cable bolt in the surrounding rock, simply aiming for a cable with large diameter and high elongation often leads to difficulties in implementing economical and effective support countermeasures for roadway stability. Therefore, it is still necessary to develop corresponding optimization measures based on the nature of the breaking of the CBFS.

This study focused on the performance and mechanism of breakage of the anchor cable under joint-plane shear action. A cable-bolt free-section strengthening device (CFSD) was designed that avoids shear damage on the cable-bolt surface, reduces shear stress concentration, and improves the shear resistance of the cable bolt. Structural optimization of the device and performance evaluation were carried out by numerical simulation, theoretical analysis, and laboratory testing. The research results can provide a reference for the development of anchor cable support technology.

## 2. Analysis of Shear Characteristics of Cable Bolt

### 2.1. Field Investigation on Cable-Bolt Failure

The Zhaojiazhai Coal Mine is located in Xinzheng City in the central region of China. The average burial depth of the 12,209 working face in the mine is 410 m, with a coal seam thickness of up to 8.5 m. The whole working face coal seam is extracted using comprehensive mechanized top coal caving technology. The haulage gateway in the working face is supported by a bolt-mesh-anchor combined support and 2 m wide high-strength concrete filling along the side of the gateway to maintain its stability for the next working face.

**Figure 1:**
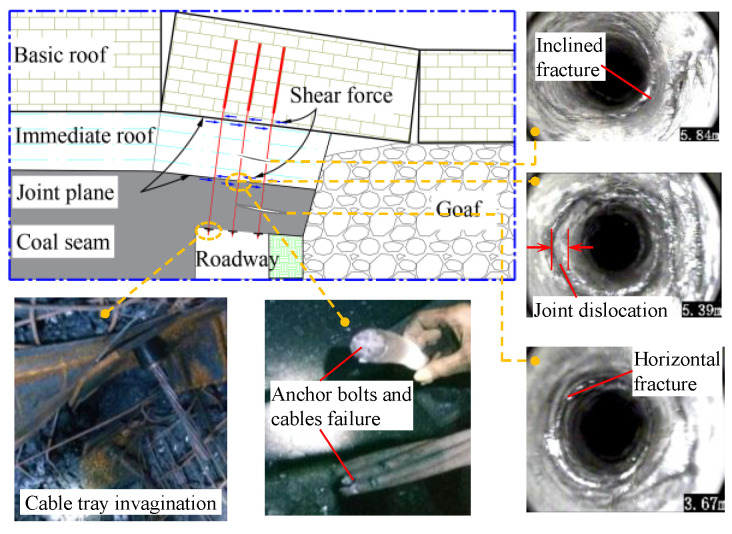
Field investigation of anchor cable failure.

During the roadway retention period, the roadway roof was relatively fragmented and deformed towards the side of the goaf (the untranslated area left after the extraction of underground coal seams). Within the larger deformation area near the goaf side, a large number of roof anchor cables broke, mostly in the unanchored free sections, with a breakage rate of about 22%. Using the ZKXG-30 borehole camera (Wuhan Tianchen Weiye Geophysical Technology Co., Ltd., Wuhan, China) to observe the deep part of the roof strata, the typical observation results are shown in Figure 1. Under the combined action of the rotary deformation of the basic roof and the limited displacement of the high-strength filling body beside the roadway, a large number of shear fractures, horizontal fractures, and joint slip phenomena occurred in the roof coal and the immediate roof near the goaf side. The dilatancy behavior of roof rock made the anchor cables produce a large axial tension, and the cable trays appeared invaginated. The probability of damage to the free section of the anchor cable increased under the combined action of tension and shear, leading to a reduction in support strength and serious threatening of the overall stability of the roadway surrounding rock.

### 2.2. Shear Failure Mechanism

**Figure 2:**
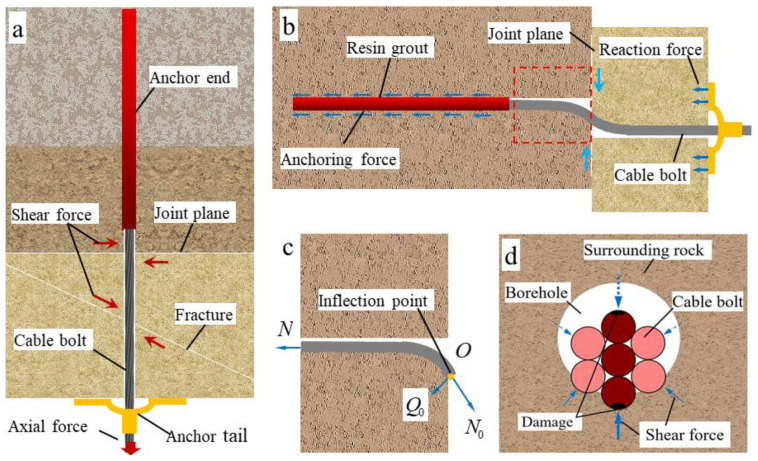
Shear characteristics of cable-bolt free section. (**a**) Shear effect; (**b**) Shear deformation; (**c**) Section of internal force at inflection point; (**d**) Shear stress distribution on cable cross-section.

In reality, cable-bolt failure is a combination of tension and shear failure [10,17,34,35]. Tensile–shear composite failure is a relatively complex mechanical process, as shown in Figure 2. In normal operation, the CBFS is axially stretched under active prestress or passive suspension to control deformation of the surrounding rock. When an unstable rock mass slides along a joint plane or fracture, the rock mass exerts a strong transverse shear effect on the CBFS, and the cable bolt is in the tensile–shear stress state, as shown in Figure 2a. Under joint-plane shear action, the cable body bends along the shear direction and compresses the rock mass on the hole wall. Owing to the weak bending capacity of the cable and cable ends that are fixed by the anchoring agent and tray, axial tensile deformation and transverse compressive rock deformation of the cable further increase with the shear displacement. The cable-bolt deformation is distributed asymmetrically along both sides of the joint plane, as shown in Figure 2b.

Half of the cable bolt along the joint plane was considered for force analysis, as shown in Figure 2c. According to the symmetry principle, the bending moment at the intersection point *O*, which is called the inflection point, is zero. The largest compressive deformation of the rock mass occurs at this point. Generally, as the shear displacement increases, the cross-sectional axial force *N*_0_ and shear force *Q*_0_ at the inflection point *O* continuously increase until the cable-bolt axial force and shear force satisfy the Tresca strength criterion [14,36,37,38] and tensile–shear failure of the cable occurs. The strength criterion is expressed as follows:

(1) $${\left(\frac{N}{N_{f}}\right)}^{2}+{\left(\frac{Q}{Q_{f}}\right)}^{2}=1$$

where *N* and *Q* are the cross-sectional tensile and shear forces, respectively, at cable-bolt failure; *N_f_* and *Q_f_* are the ultimate tensile strength and ultimate shear strength of the cable bolt, respectively. From Equation (1), the shear force that a cable bolt can bear is negatively correlated with its axial force. Owing to the high pre-tightening force and continuous increase of the cable axial force in the joint-plane shear action, the cable shear strength continues to diminish, and the probability of tensile–shear failure increases.

Most cable-bolt types consist of multiple high-strength steel wires wrapped together. Under axial tension, the tensile properties of each steel wire can be better exploited, but this structure negatively affects the cable bolt under lateral shear.

A shear stress distribution diagram of the cross-section at the inflection point of a sheared cable bolt is shown in Figure 2d. When shear displacement of the joint plane increases, the steel wires at the upper and lower ends of the cable body first contact and interact with the hole-wall rock mass. The compression displacement of these wires is largest compared with that of other steel wires, resulting in maximum reverse resistance of the hole-wall rock mass, which leads to shear stress concentration at the outer edge of the steel wires. The shear force is transmitted between the steel wires of the cable bolt through the contact point, which is unstable. Because of the small lateral constraint of the surrounding steel wires, the wires constantly adjust during shear transfer. The final cross-sectional shear stress is mainly concentrated in the three steel wires at the upper and lower ends and the center.

According to Equation (1), the steel wire with larger shear stress first satisfies the tensile–shear failure condition and then breaks, which further exacerbates evolution of the broken strand and causes failure of the entire cable bolt; in other words, the shear transfer mode of this section exacerbates tensile–shear failure of the cable bolt.

## 3. Strengthening Method and Design of Cable-Bolt Free-Section Strengthening Device

### 3.1. Introduction to Cable-Bolt Free-Section Strengthening Device

**Figure 3:**
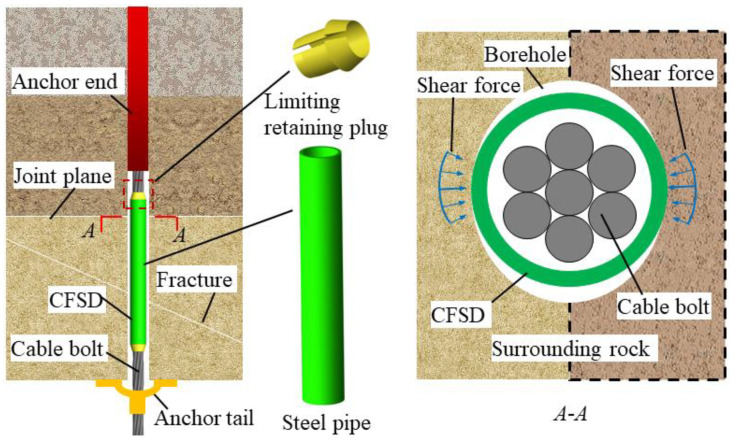
Structure of cable-bolt free-section strengthening device (CFSD) and sketch showing its application in a borehole.

According to the cable-bolt shear failure characteristics, effectively avoiding a harmful stress concentration is an effective approach to reducing the probability of cable-bolt shear failure. To improve the shear strength of a cable bolt, fully utilize its axial bearing capacity, and enhance the shear resistance of surrounding rock, an optimization method using a high-strength steel pipe to protect the sheared CBFS is proposed; accordingly, a CFSD was designed, as shown in Figure 3.

The main CFSD structure consists of a limiting retaining plug and steel pipe. The limiting retaining plug is made of rubber and has the role of fixing the steel pipe. The plug body opening has an inner diameter that is slightly larger than the anchor diameter. The plug head is exposed at both ends of the slotted pipe; its maximum outer diameter is equal to the pipe diameter and gradually decreases to the end, which is convenient for pushing into the borehole. The pipe is made of high-strength steel, and its section size is limited by the diameters of the borehole and cable bolt. Therefore, the strength and size of the CFSD should be adapted to the specific cable bolt. In practical engineering applications, a limiting retaining plug and slotted pipe are inserted into the cable bolt in turn after drilling the borehole. The flexible plug is pressed to embed it into the gap between the steel pipe and cable bolt, such that the device can be firmly fixed on the CBFS, and is then pushed into the borehole with the cable bolt to ensure stability at a potential sliding structural plane.

The CFSD mechanism is twofold: (i) the steel pipe is completely wrapped in the shear section of the CBFS to avoid direct interaction between the exposed steel-wire bundle and the hole-wall rock mass, which can effectively reduce the stress concentration of the anchor wire bundle and avoid shear damage of the outer wires; (ii) the cable bolt’s transverse shear strength changes from the original cable section strength alone to the sum of the shear strength of the steel-pipe section and cable-bolt section, and the cable bolt’s shear capacity is improved, alleviating the contradiction between higher cable axial tension and lower shear capacity.

### 3.2. Parameter Design

**Figure 4:**
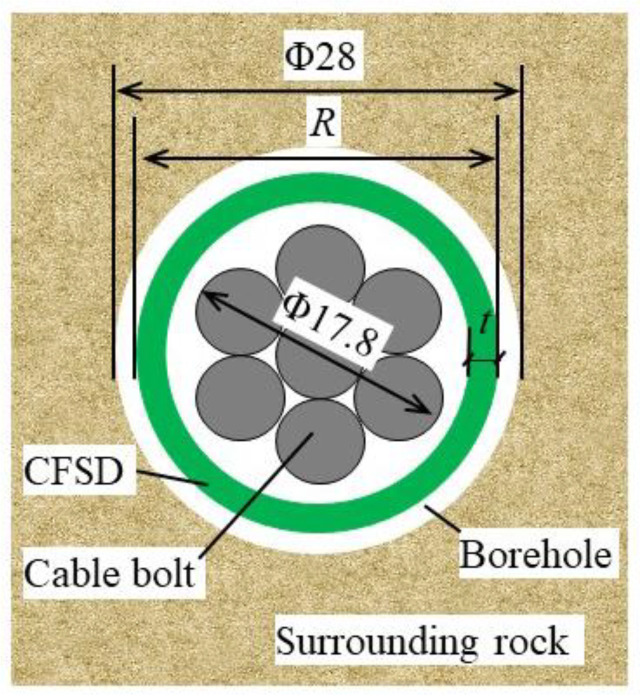
Positional relationship of device in borehole.

A 17.8 mm anchor cable was employed as the object of investigation, and the CFSD was designed to accommodate this. The cable bolt consists of seven steel strands, and the borehole diameter is typically 28.0 mm. Figure 4 shows the positional relationship between the cable bolt and the device in the borehole. The ultimate shear force of the cable bolt with the CFSD can be expressed as follows:

(2) $$Q_{f}{}_{s}=\tau_{f1}A_{b}+\tau_{f2}\pi (R-t)t$$

where *Q_fs_* is the ultimate shear force of the cable bolt with the CFSD, *τ_f_*_1_ is the cable-bolt shear strength, *τ_f_*_2_ is the steel-pipe section shear strength, *A*_b_ is the cable-bolt cross-sectional area, *R* is the steel-pipe outer diameter, and *t* is the wall thickness. From Equation (2), increasing the steel-pipe thickness can improve the ultimate shear bearing capacity of the strengthened cable bolt, but the wall thickness is limited by the borehole and cable-bolt diameter. According to the geometrical relationship of each structural section shown in Figure 4, the allowable wall thickness range is 1–5 mm. Considering the buffer effect of the device and the convenience of pushing the device into the borehole, the outer diameter of the steel pipe was set as 25 mm, and the wall thickness was set as 2 mm.

According to the operating mechanism of a CFSD, design of the mechanical parameters of the steel pipe should follow the principle that the CFSD structure is complete without failure before the cable bolt breaks under the protection of the strengthening device in the shearing process. On this basis, steel pipes with large cross-sectional shear strength are selected to improve the transverse shear resistance of the cable bolt. Comprehensively considering the working principle and economic cost of the CFSD, we selected #45 high-strength steel pipe, which is a mature technology with wide use in the Chinese domestic market, as the material for the strengthening device to meet the needs of this research and future practical application. The chemical composition of #45 steel includes a carbon (C) content of 0.42–0.50%, a silicon (Si) content of 0.17–0.37%, a manganese (Mn) content of 0.50–0.80%, and a chromium (Cr) content of ≤0.25%. The design parameters of the strengthening device are shown in Table 1.

According to tests on anchorage joints [38,39], the transverse shear deformation section length of rock bolts and cable bolts near the joint plane is approximately two-to-four times their diameter; the shear deformation section of non-anchorage bolts will be longer. Considering the plug installation length and construction operation error, the control length of the CFSD for a single joint plane was determined as 200 mm. In practical applications, the CFSD design length is equal to the loose circle test depth in a coal-mine roadway. If necessary, the strengthening device can be arranged along the entire CBFS to ensure effective control of potential slipping of the structural plane.

**Figure 5:**
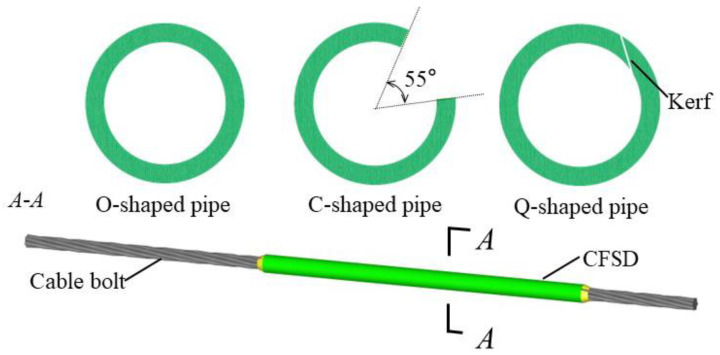
Different types of CFSD sections.

Different steel-pipe section types for the CFSD have different effects on improving the shear capacity of the cable bolt. Three steel-pipe section types were considered in the preliminary design: type O, type C, and type Q (Figure 5). The O-shaped pipe (O-CFSD) was a steel pipe with a continuous wall. The C-shaped pipe (C-CFSD) was an open steel pipe with an opening center angle of 55°, such that the pipe wall was completely closed at the opening under the shear effect. The Q-shaped pipe (Q-CFSD) was a slotted steel pipe; the wall is cut along the longitudinal direction, and the kerf is inclined, such that the wall moves in the opposite direction along the kerf under shear action to wrap the cable bolt.

## 4. Performance and Type Selection Simulations

### 4.1. Models and Schemes

**Figure 6:**
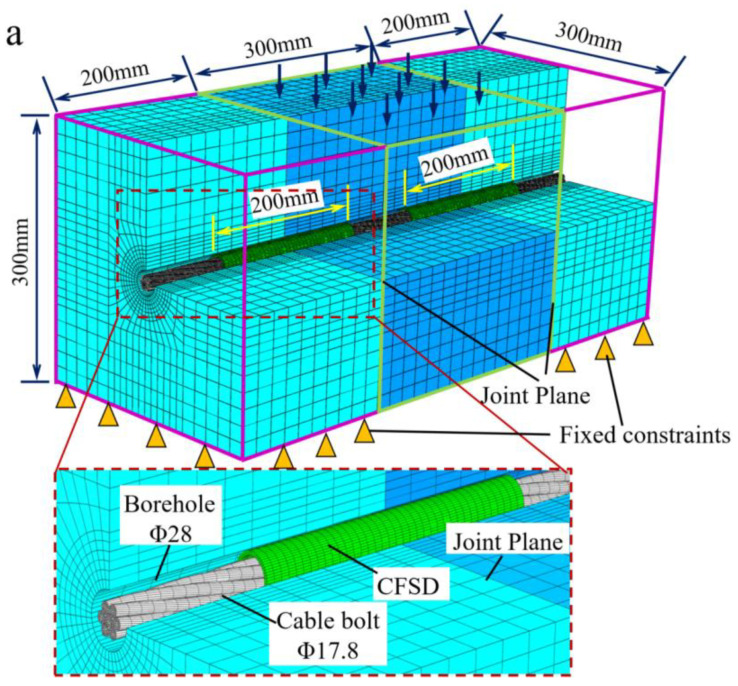

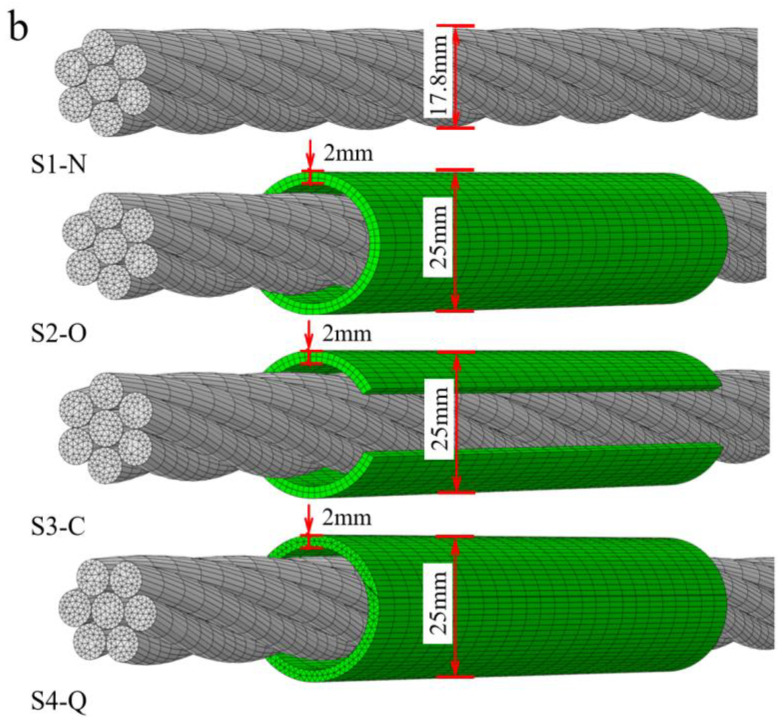
Cable-bolt double-shear numerical experiment. (**a**) Experimental model with CFSD; (**b**) Experimental specimen models. S1-N represents the specimen model for the bare cable bolt (without CFSD); S2-O, S3-C, and S4-Q represent the specimen models for the cable bolt with the O-, C-, and Q-shaped CFSDs, respectively.

To analyze the CFSD working performance and determine the optimal design, ABAQUS/Explicit finite-element analysis software was used to simulate double-shear testing of cable bolts, onto which the three types of devices were installed. The results were compared with those obtained by a double-shear test on bare cable bolts (without CFSD). The numerical model and experimental schemes are shown in Figure 6.

The model shown in Figure 6a had a size of 700 mm × 300 mm × 300 mm and consisted of three coplanar rock blocks. The width of the middle rock block was 300 mm, and the widths of the rock blocks on both sides of the model were 200 mm. A borehole with a diameter of 28 mm was set along the longitudinal direction in the center of the rock masses. The contact surface between the three rock blocks simulated the joint-plane shear effect by setting a normal hard contact, and the tangential friction coefficient to 0.3. A 17.8 mm diameter seven-strand steel cable bolt was set at the borehole center perpendicular to the joint plane. The cable bolt was not in contact with the borehole wall to simulate CBFS. The interactions between the cable bolt and hole-wall surface in this research were set to a friction coefficient of 0.1 [40]. According to the design results for the CFSD, the device model length was 200 mm, the outer diameter was 25 mm, the wall thickness was 2 mm, and the device was symmetrically set along the joint plane, as shown in Figure 6a. Each steel strand was divided into independent grids, and the lay length of the cable bolt was 260 mm, which is consistent with that of the actual cable bolt.

The loading process was completed in two analysis steps. The parameter settings are listed in Table 2. Fixed boundary conditions were set on the bottom rock block surface on both sides of the model and the outer-end surface of one of the rock blocks, including the anchor cable; the remaining model boundaries were free. Normal pressure of 1 MPa was applied to the outer-end surface of the rock on the other side. The purpose was to obtain the shear force during the free-sliding stage of the rock joint planes, while reducing its contribution to the joint-plane shear force during the anchor cable reinforcement stage, thereby highlighting the resistance-increasing effect of the strengthening devices and cable bolts. Axial tension of 90 kN was applied by longitudinally stretching the anchor cable with a displacement of 1.7 mm, and the tensile displacement was fixed during the shearing process to maintain a tensile working state. A vertical load controlled by displacement was applied to the top of the middle rock block. The loading speed was 0.2 mm/s to simulate the entire process of joint-plane shearing. The reaction force on the load surface and the cable-bolt axial force were monitored during the shear process.

Numerical simulations of double-shear experiments on a bare cable bolt (without CFSD) and cable bolts with O-, C-, and Q-CFSDs were carried out. The simulation schemes are shown in Figure 6b.

### 4.2. Model Parameters

The model material consisted of the rock blocks, cable bolt, and steel pipe. The Mohr–Coulomb strength criterion was adopted for characterizing the failure mechanism of the rock blocks [41]. The mechanical parameters were determined based on the laboratory test results of the rock samples obtained from the Zhaojiazhai coal mine, and the lithology of the rock samples was sandy mudstone. The cable bolts and steel pipes are both metallic materials, for which the von Mises failure criterion is more applicable and was therefore adopted in these simulations [19]. The mechanical parameters of steel pipe were determined based on measured data for the #45 steel pipe. The mechanical properties of the rock and steel-pipe models are listed in Table 3.

**Figure 7:**
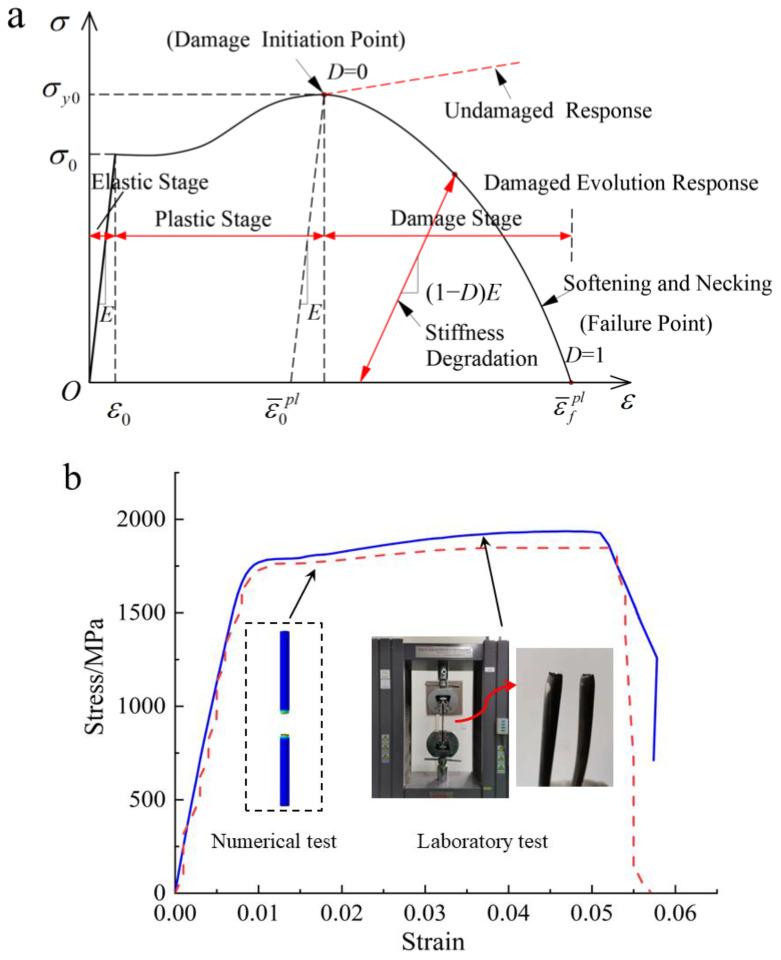
Numerical simulation of tensile failure process of steel strand. (**a**) Steel strand material property model; E: Young’s modulus of the material; $\varepsilon_{0}$: ultimate elastic strain; ${\bar{\varepsilon }}_{0}^{pl}$: ultimate plastic strain during plastic stage; ${\bar{\varepsilon }}_{f}^{pl}$: fracture strain; *D*: damage variable; $\sigma_{0}$: elastic limit stress; $\sigma_{y0}$: peak stress. (**b**) Comparison of steel strand stress–strain curves.

To describe the entire shear failure process of the cable bolt, elastic, plastic, ductile, and shear damage models were used to simulate the steel strands [42]. Based on the tensile test and evolution of damage results for the steel, the complete stress–strain curve of the anchor cable in uniaxial tension can be divided into three stages: the elastic, plastic, and damage stages, as shown in Figure 7a. In the elastic stage, the stress increases linearly with strain, with a slope equal to Young’s modulus. In the plastic stage, after reaching the elastic limit strain, the stress increment is relatively small, while the strain continues to increase, resulting in nonlinear stress–strain behavior. If a ductile damage model is not considered, the material’s strength gradually increases, and the stiffness remains constant in the plastic stage. In the damage stage, the strain reaches the ultimate plastic strain, the Young’s modulus of the material gradually decreases, and the strength continuously decreases, causing the occurrence of necking. As the damage variable increases to 1, the material strength gradually reduces to zero, and it undergoes tensile failure when the strain reaches the fracture strain.

In order to obtain the model parameters of the cable bolt, the uniaxial tensile test of the steel strands was carried out. A section of steel wire with a length of 200 mm was arbitrarily intercepted as a tensile sample from a cable bolt with a diameter of 17.8 mm. The stress–strain curve of the cable steel strand was obtained by tensile testing using a WDW-100 universal testing machine (Jinan Yingbaixin Testing Instrument Co., Ltd., Jinan, China) (Figure 7b). ABAQUS software was used to simulate the tensile test under the same conditions on the numerical cable steel strand model. The stress–strain curve obtained from the numerical simulation was compared with the constitutive curve obtained from laboratory tests, as shown in Figure 7b. The constitutive curves obtained from both methods are in good agreement, validating the correctness of the input parameters for the steel strand’s tensile damage model. Owing to lack of experimental data, such as pure shear test data for the steel strands, some model parameters were determined using a trial-and-error method to obtain reasonable values. The properties of the cable strand material are shown in Table 4.

### 4.3. Simulation Results and Analysis

**Figure 8:**
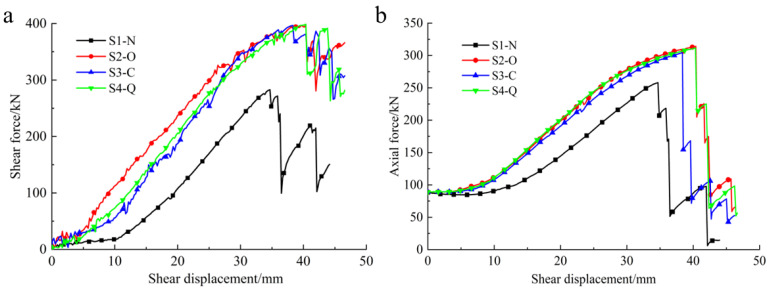
Curves of numerical experiment results. (**a**) Joint-plane shear force–shear displacement curves; (**b**) Cable-bolt axial force–shear displacement curves.

Figure 8 shows the joint-plane shear force–shear displacement and cable-bolt axial force–shear displacement curves obtained from the double-shear numerical experiments on cable bolts with different CFSD types. Statistics for the strength parameters and increments in the numerical simulation results are shown in Table 5.

As indicated by the joint-plane shear force–shear displacement curves in Figure 8a, in the double-shear test on anchor cable samples with different structures, the joint-plane shear force exhibited the same trend, with three stages in the process: constant shear force generated by free sliding of the joint plane, rapid increase of the shear force induced by the added resistance of the cable bolt, and shear force reduction caused by cable-bolt failure. Owing to the presence of the cable bolts, the peak shear force was much larger than the frictional resistance of the free-sliding joint surface, indicating that the CBFS significantly contributed to inhibition of the joint-plane slip and cannot be ignored. The shear forces of the cable bolts with the CFSDs (S2-O, S3-C, S4-Q) were higher than that of the bare cable bolt (S1-N) under the same shear displacement. As shown in Figure 8a and Table 5, the peak shear forces for specimens S1-N, S2-O, S3-C, and S4-Q were 283 kN, 397 kN, 396 kN, and 399 kN, respectively. Compared with that of the bare anchor cable specimen (S1-N), the peak shear forces for specimens S2-O, S3-C, and S4-Q increased by approximately 40%, which reflects the significant improvement of the CFSD on the shear resistance of the CBFS. Moreover, there was little difference in enhancing the shear strength of the cable bolt between O-shaped, C-shaped, and Q-shaped CFSDs.

As shown by the cable-bolt axial force–shear displacement curves in Figure 8b, an axial force was synchronously generated during shear of the cable bolt and increased with the shear displacement. Once the axial force peaked, the anchor cable steel strands broke one by one, causing the axial force to rapidly decrease in a step-like manner. Therefore, the shear displacement corresponding to the peak value is the ultimate shear displacement of the cable bolt. During the period of increase in the cable axial force, the axial forces of the cable bolts with CFSDs were higher than those of the bare cable bolts under the same shear displacement, and the improvements and size were approximately the same. The maximum axial force for specimen S1-N was 258 kN, while those of specimens S2-O, S3-C, and S4-Q were 314 kN, 304 kN, and 312 kN, respectively. This is an increase of approximately 20% (Table 5), which is less than the ultimate tensile load of 355 kN. As shown in Figure 8b and Table 5, the ultimate shear displacements for specimens S1-N, S2-O, S3-C, and S4-Q were 34.7 mm, 40.4 mm, 38.4 mm, and 40.3 mm, respectively. Therefore, these different types of CFSDs effectively improved the shear resistance of the cable bolt and enhanced the anti-deformation ability to better promote the axial load transfer. The optimal cable-bolt designs are the O-CFSD and Q-CFSD.

**Figure 9:**
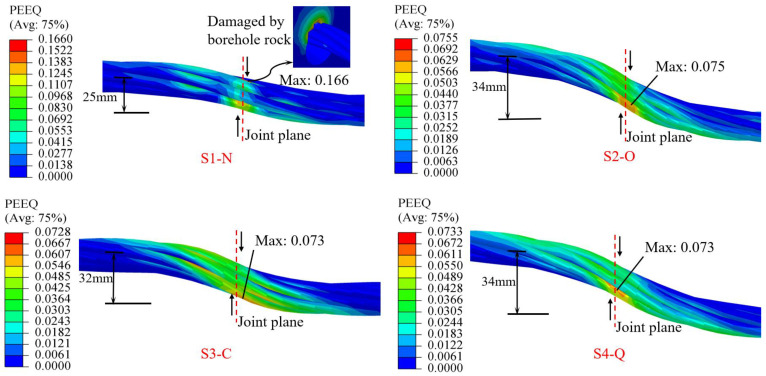
Equivalent plastic strain distributions of cable bolts for different specimens near joint plane at ultimate shear displacement. PEEQ: cumulative equivalent plastic strain in ABAQUS software.

Distributions of the equivalent plastic strain for cable bolts near the joint plane at ultimate shear displacement are shown in Figure 9. When a CBFS is sheared by the joint plane, the cable body near the inflection point is in a tensile–shear state. The transverse shear effect of the cable bolt is more significant because of the strong binding force of the rock mass, and the cable cross-section bears a large shear force. Under the combined action of axial and shear forces, the cable bolt first reaches the yield state at the inflection point, and further tensile–shear failure occurs. As shown in Figure 9, the plastic zone for all specimens at the shear limit was concentrated near the inflection point, and the maximum value of the equivalent plastic strain also fell within the inflection point. The ultimate strength of these devices is much smaller than that of the cable bolts, so setting of the CFSDs does not change the anchor cable tensile–shear failure mode. Figure 9 shows that the maximum equivalent plastic strain of specimen S1-N was 0.166, and the plastic zone was mainly concentrated on the outer-edge wires at the inflection point. The outer edge of the anchor cable strand directly interacted with the hole-wall rock, causing the stress to be concentrated on the outer-edge strands, so these strands were first to reach the yield state at the inflection point. After setting the CFSDs, the maximum equivalent plastic strains of specimens S2-O, S3-C, and S4-Q were reduced to 0.076, 0.073, and 0.073, respectively, and the plastic area significantly increased. This trend shows that the CFSDs improved the shear state of the cable-bolt structures and mobilized the cable steel strands to play a shear resistance role. Furthermore, direct squeezing of the rock mass by the cable bolt is avoided under protection of the strengthening device, so the degree of equivalent plastic strain concentration is reduced.

Additionally, shear resistance of the cable bolt is exerted by both transverse shear deformation and axial tensile deformation [33]. The greater the lateral shear deformation, the greater is the shear resistance provided by the cable bolt. Figure 9 shows that specimen S1-N had the smallest lateral shear deformation of 25 mm. After setting the strengthening device, the transverse shear deformations of specimens S2-O, S3-C, and S4-Q increased to 34 mm, 32 mm, and 34 mm, respectively. This result showed that CFSDs can effectively increase the shear strength of the anchor cable system. Comparison of the different types of CFSDs showed that the Q-CFSD design exhibited the best reduction of equivalent plastic strain concentration, thereby enhancing the shear strength of the anchor cable system and improving deformability.

**Figure 10:**
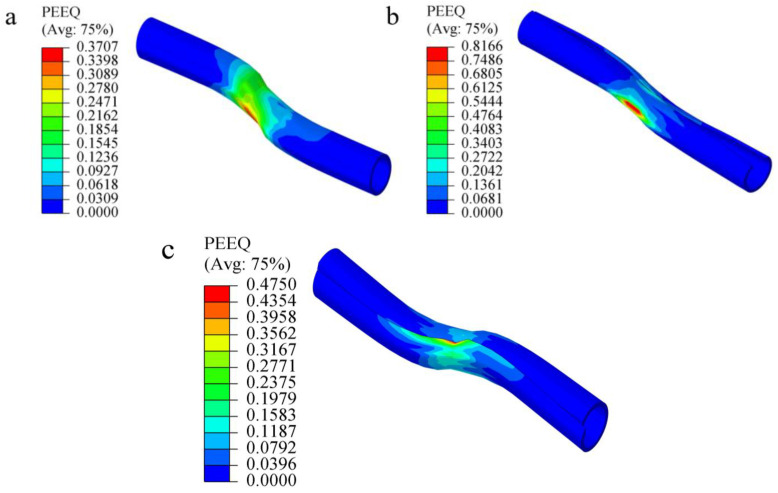
Distributions of equivalent plastic strain of CFSDs at ultimate shear displacement. (**a**) O-shaped cable-bolt free-section strengthening device; (**b**) C-shaped cable-bolt free-section strengthening device; (**c**) Q-shaped cable-bolt free-section strengthening device.

Distributions of the equivalent plastic strain for the different types of CFSDs at ultimate shear displacement are shown in Figure 10. Figure 10a shows that the plastic zone of O-CFSD was mainly concentrated on the pipe wall near the inflection point, and the maximum equivalent plastic strain was 0.371, located where the pipe body is in contact with the rock mass. The complete pipe body of O-CFSD is prone to failure before the cable bolt breaks. Figure 10b shows that the plastic zone of C-CFSD was also concentrated on the pipe wall near the inflection point; however, the maximum equivalent plastic strain was 0.817, located at the bottom of the pipe in direct contact with the rock mass, which may lead to localized damage of the pipe. Figure 10c shows that the plastic zone of Q-CFSD was mainly concentrated on the edge of the cut at the inflection point. The maximum equivalent plastic strain was 0.475, located in a small area at the edge of the pipe wall at the seam. The plastic strain in the rest of the pipe-wall area was very small, which ensured that the CFSD structure did not fail before shear break of the cable bolt. The CFSDs produced both lateral deformation and axial tensile deformation with the cable bolts to bear the transverse shear action. The O-CFSD was a complete structure and had poor ability to adapt to deformation, so it produced large plastic deformation in the pipe body. C-CFSD and Q-CFSD both had penetrating kerfs, which increased the degrees of freedom, improved the deformation ability of the device, and readily deformed together with the anchor cable. In contrast, Q-CFSD had better adaptability to deformation.

**Figure 11:**
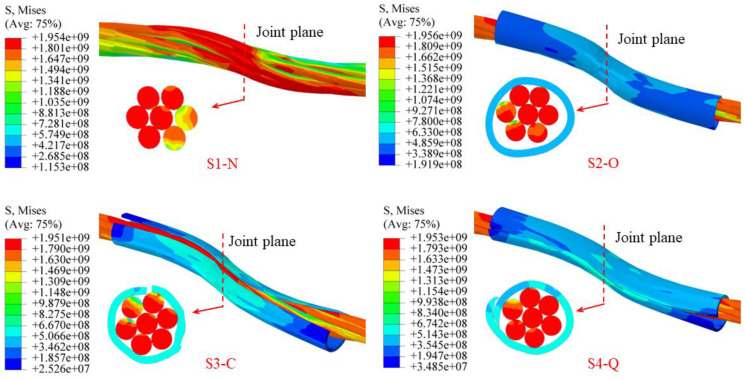
Stress distributions of specimen models near joint plane at ultimate shear displacement. S, Mises: equivalent (Mises) stress (Pa).

Figure 11 shows the equivalent stress distributions of the specimen models near the joint plane at ultimate shear displacement. The maximum equivalent stress at the inflection point of the specimens at the shear limit reached the ultimate strength values of the materials (cable bolt: 1950 MPa; CFSD: 600 MPa). The stress distribution of each steel strand was uneven on the cross-section at the inflection point for specimen S1-N, and the average cross-sectional stress of the steel strand was far from its strength limit. This shows that the shear resistance of specimen S1-N was not fully exerted. However, the stress distributions of each steel strand for specimens S2-O, S3-C, and S4-Q were uniform, and the average cross-sectional stress of most strands reached or approached their strength limit, which indicated that the shear resistance of the cable bolts was well exerted. Comparing the working effects of different types of CFSDs (Figure 11), we found that the pipe body of O-CFSD simultaneously produced lateral extrusion deformation and axial tensile deformation during the shearing process, and was not coordinated with the anchor cable deformation, so each part contributed to the shear resistance. The C-CFSD was deformed by transverse extrusion, with the pipe wall being closed along the kerf, which tightly fitted the anchor cable and applied an inward compressive stress. The C-CFSD pipe body was bent and deformed together with the cable bolt: the lack of lateral binding force at both ends of the steel pipe caused the pipe wall to open. The Q-CFSD was deformed by transverse extrusion, and the pipe wall rolled inward along the kerf to tightly wrap and squeeze the cable body. Simultaneously, the pipe walls moved with each other in the axial direction to adapt to the axial tensile deformation. The Q-CFSD had strong deformability, and deformation was coordinated with the anchor cable during the shearing process to form a pipe–cable bearing unit. Lateral confinement of the Q-CFSD pipe wall on the steel-wire bundle was higher than that of C-CFSD, so the Q-type pipe–cable unit had the highest integrity. In addition, because of the large width of the C-CFSD pipe kerf, relative movement of the pipe wall was blocked when the opening position was toward the shear sliding direction of a rock mass, so C-CFSD could not form a grip around the cable body. In the case of uncertain shear direction of a rock mass in field applications, the exact installation orientation of the C-CFSD opening cannot be guaranteed. The Q-CFSD clearly demonstrated the best working performance.

Results of the double-shear experimental simulation with different types of cable-bolt structures revealed that the CFSD significantly improved the shear resistance and deformation resistance of the anchor cable system, while protecting the cable bolt and avoiding harmful stress damage. Through comprehensive comparison, the Q-CFSD was determined as the optimal section type.

## 5. Laboratory Experiments

### 5.1. Experimental Devices and Process

**Figure 12:**
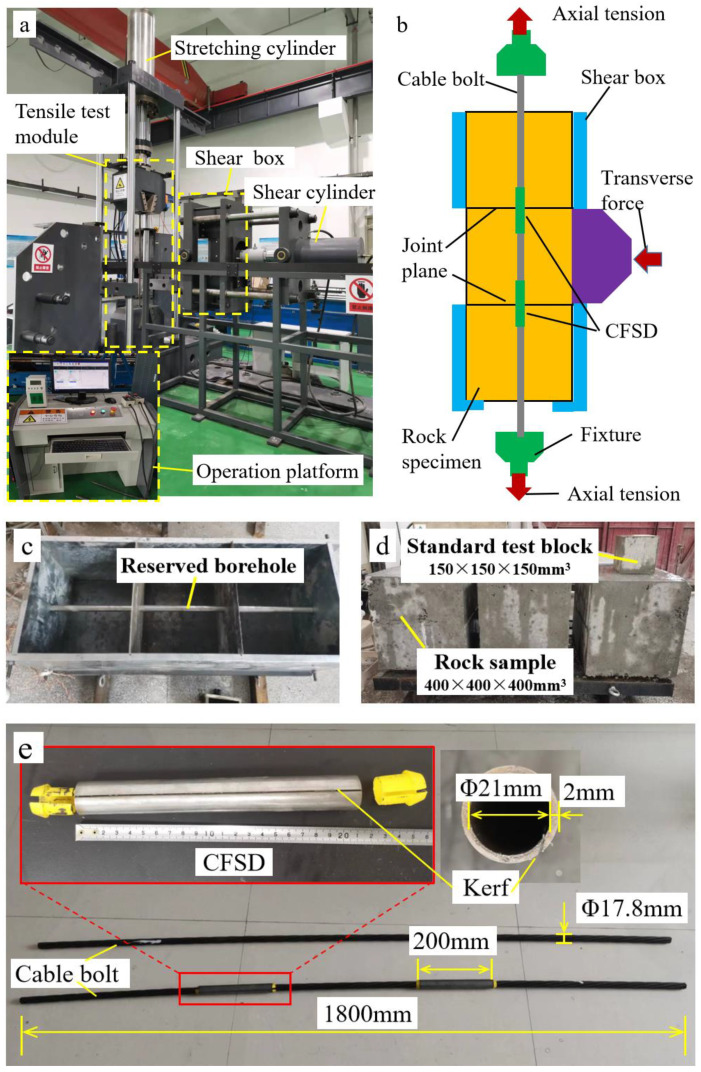
Double-shear experimental equipment and materials. (**a**) Comprehensive experimental equipment for roadway support materials; (**b**) Working principle of double-shear test; (**c**) Sample mold and steel pipe for prefabricated anchor holes; (**d**) Rock sample and standard test block; (**e**) Cable bolt and cable bolt with Q-CFSD used in double-shear experiment.

To verify the operation of the CFSD, the cable-bolt double-shear experimental equipment shown in Figure 12a was developed. The equipment consists of vertical and horizontal loading systems; the two systems are coupled by a computer control platform. The principle of the double-shear experiment is illustrated in Figure 12b. Three rock specimens, matching the shear box size, with boreholes in the middle were prepared before the test and placed vertically in the shear box to ensure alignment of the boreholes. The shear box consisted of four reaction beams and two support steel plates hollowed out in the middle. The loading plate of the horizontal loading system could pass through the middle of one of the steel plates to load an intermediate rock specimen in the horizontal direction. Another hollowed-out steel plate imposed fixed constraints on the upper and lower rock specimens in the horizontal direction, but did not limit the rock specimens loaded in the middle. The experimental cable bolt was first inserted into the borehole at the middle of the rock specimen, and the horizontal loading system and shear box were then pushed into the vertical loading system along the support. Finally, the fixtures clamped both ends of the cable bolt in the vertical loading system, such that axial and lateral loads of the anchor cable could be achieved. Thus, the cable bolt was loaded in both the axial and lateral directions.

The rock specimens used in the experiments were prepared using specific simulated materials, namely, cement, sand, pebbles, and water in the mass ratio of 1:1.29:2.4:0.36. The concrete was prepared in a mold matching the shear box size. The mold consisted of three compartments with a size of 400 mm × 400 mm × 400 mm, which could produce three specimens in one preparation. A steel pipe with a diameter of 28 mm was inserted into the middle of the mold, such that the formed specimens had a borehole with a diameter of 28 mm (Figure 12c). The simulated materials with a specific proportion of surrounding rock were poured into the mold. The rock specimens were cured for 28 days, and the mold was removed after the first day. A small specimen (150 mm × 150 mm × 150 mm) was also prepared using a standard cube mold under the same conditions, and its uniaxial compressive strength was measured as 58.6 MPa using a digital dynamometer (Figure 12d).

The cable bolt alone and cable bolt with the Q-CFSD used in the double-shear experiments are shown in Figure 12e. The Q-shaped pipe in the CFSD was a #45 high-strength steel pipe cut obliquely along the length direction, such that the pipe walls on both sides of the kerf formed a 20° wedge angle. The length of a single CFSD was 200 mm, the outer diameter was 25 mm, and the wall thickness was 2 mm. The experimental cable bolt was of 17.8 mm diameter seven-strand steel with a length of 1800 mm. Before the double-shear experiment on the cable bolt with the CFSDs, installation of the CFSDs was carried out in the following steps: the slotted steel pipes were first inserted into the cable bolt, and limiting retaining plugs were used to fix these at the corresponding positions of the two joint planes; the slotted steel pipes were then pushed into the reserved borehole of the rock specimens that were stacked vertically in the shear box along with the cable bolt (Figure 12b).

To more accurately test operation of the CFSD, double-shear experiments on the bare cable bolt (without CFSDs) and cable bolt with CFSDs were carried out. The test loading method proceeded as follows: first, a 100 kN axial load was applied in the vertical direction, and the displacement was kept constant; a 1 mm/min velocity load was then applied in the horizontal direction to break the cable bolt.

### 5.2. Experimental Results and Analysis

**Figure 13:**
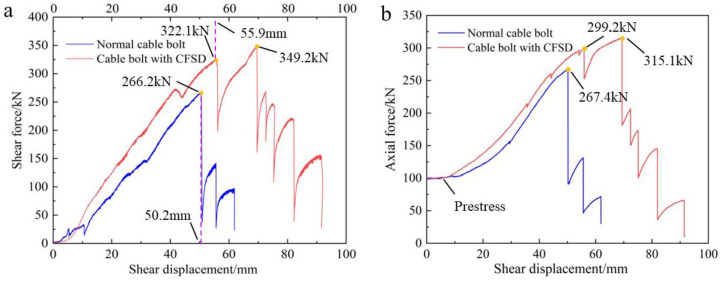
Experimental monitoring results. (**a**) Joint shear force–shear displacement curves of normal cable bolt and cable bolt with CFSD; (**b**) Axial force–shear displacement curves of normal cable bolt and cable bolt with CFSD.

Figure 13 shows the joint-plane shear force–shear displacement curves and cable-bolt axial force–shear displacement curves, according to the monitoring data collected during the double-shear experiments. As shown in Figure 13a, the shapes of the joint-plane shear force–shear displacement curves of the bare cable bolt and cable bolt with the CFSDs are essentially identical. However, at the stage where the cable bolt’s resistance against the joint-plane shear force increased, the joint-plane shear force of the cable bolt with the CFSDs was larger than that of the normal anchor cable without the CFSDs under the same shear displacement. The peak shear force on the joint planes of the normal cable bolt was 266.2 kN, while that of the cable bolt with the CFSDs was 349.2 kN, marking an increase of 31%. The ultimate shear displacement of the normal cable bolt was 50.2 mm, while that of the cable bolt with the CFSDs was 55.9 mm under joint-plane shear action, marking an increase of 11%. The change of the shear force curves on the joint plane confirmed that the CFSD could increase the shear strength of the anchor cable system and improve the ability of the normal cable bolt to resist shear deformation.

Figure 13b shows that, under joint-plane shear action, the cable bolts simultaneously generated axial tension. In the post-peak stage, as the steel strands broke one by one, the axial force of the anchor cable exhibited a step-like decrease pattern, similar to the numerical simulation results. When the anchor cable increased the joint-plane shear force, the axial force of the cable bolt with the CFSDs was larger than that of the normal cable bolt under the same shear displacement: the peak axial force of the normal cable bolt was 267.4 kN, while that of the cable bolt with the CFSDs was 315.1 kN, marking an increase of 18%. Notably, the peak axial force of the cable bolt with the CFSDs was reached after the first wire in the cable bolt broke, and the axial force at the ultimate shear displacement was 299.2 kN. Therefore, the CFSD could improve the axial force of a broken-wire cable bolt. Owing to the transverse deformation of the CFSDs, the device rolled inward, and radial pressure was exerted on the cable bolt, such that the cable bolt was still highly capable of transmitting the axial force even after a wire had broken.

**Figure 14:**
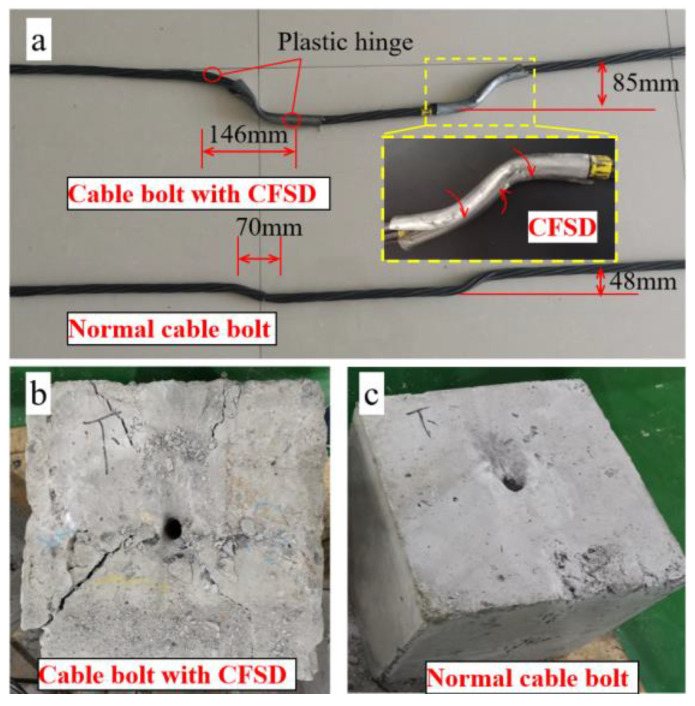
Photographs of cable bolts and rock orifices after double-shear experiment. (**a**) Shear deformation of cable bolts and CFSD; (**b**) Failure characteristics of rock orifice under cable bolt with CFSD shear action; (**c**) Failure characteristics of rock orifice under bare cable-bolt shear action.

Characteristics of the cable-bolt deformation and orifice failure after the double-shear experiment are shown in Figure 14. Figure 14a shows that lateral deformations of the normal cable bolt and cable bolt with the CFSDs were 48 mm and 85 mm, respectively, and the horizontal distances of the plastic hinge were 70 mm and 146 mm, respectively, indicating that the lateral shear capacity of the cable system and its ability to resist shear deformation significantly improved after adding the CFSDs. The CFSD deformation state after the shearing action of the joint plane was S-shaped, and its surface was covered with scratches where it interacted with the hole-wall rock mass. This indicates that the CFSD effectively protected the cable bolt against surface damage. Because the middle part of the devices was squeezed by the hole-wall rock mass, the pipe walls rolled inward along the kerf and tightly wrapped the cable bolt to form a bearing whole. This indicates that the CFSDs are very capable of adapting to cable-bolt deformation, and their strength matches the cable-bolt tension–shear composite bearing capacity. Most importantly, the CFSD modified the shear force transmission mode on the joint plane by changing the point-to-point transmission by a single steel wire to a circumferential extrusion effect exerted by the pipe wall, which fully stimulated the shear resistance of each cable-bolt steel wire.

Figure 14b shows that, after the double-shear experiment on the cable bolt, the failure state at the orifice of the rock mass was horn-shaped, and an X-type failure crack appeared on the joint plane after shearing the cable bolt with the CFSD. Additionally, the degree of damage was significantly greater than that of the orifice with the normal cable bolt under shear action. As the degree of damage to the rock mass at the orifice is proportional to the work done by the shearing action, the greater the degree of damage to the rock mass at the orifice, the more work was absorbed by the anchor system in the interaction between the anchor cable and the rock mass, and the stronger the shear resistance capacity of the anchor system. Therefore, the cable bolt with the additional CFSDs had greater resistance to slip deformation of the jointed rock mass under the same rock strength, which is beneficial for controlling the slip of a jointed rock mass using an anchor cable system.

## 6. Discussion

An unstable rock mass in a roadway loose zone slides along the structural plane. When the displacement is small, the rock mass has not yet contacted the anchor cable structure, and the CFSD will not work. At this stage, the rock mass can be restrained from sliding by prestressing the anchor cables to increase the normal stress of the joint plane.

**Figure 15:**
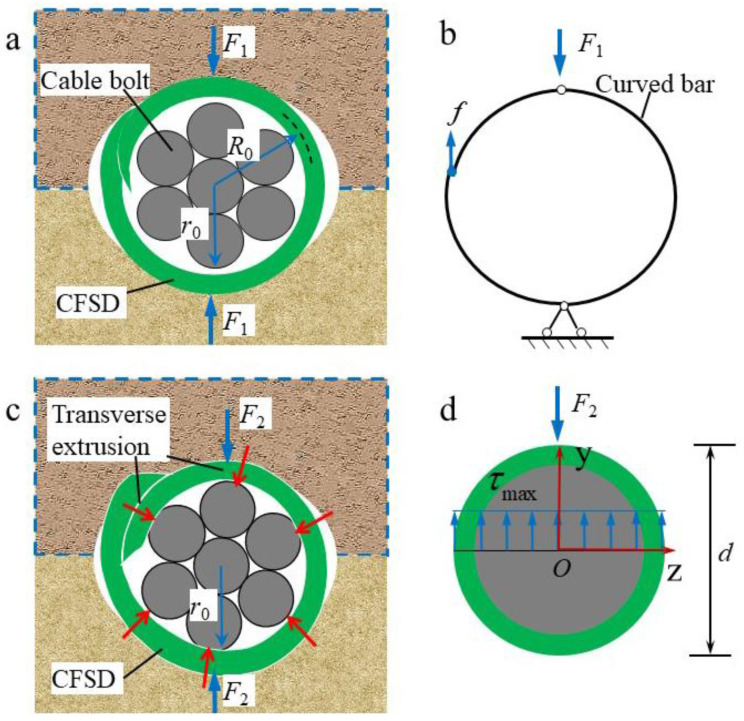
Working mechanisms of cable bolt with CFSD in two stages during shear action of joint plane. (**a**) Schematic diagram of CFSD shear resistance at shrinkage stage. *F*_1_: shear resistance provided by CFSD at shrinkage stage; *r*_0_: cable-bolt radius; *R*_0_: average radius of slotted pipe. (**b**) Two-hinged semicircular arch model of CFSD at shrinkage stage. (**c**) Schematic diagram of shear resistance provided by pipe–cable bearing whole; *F*_2_: shear resistance provided by pipe–cable bearing whole. (**d**) Solid circular beam model of entire pipe–cable bearing; $\tau_{\max}$: maximum shear stress on pipe–cable bearing whole section; *d*: pipe–cable bearing diameter.

The shear displacement continues to increase, and the hole-wall rock compresses the slotted steel pipe, such that the pipe wall moves in the opposite direction along the incision, and the steel pipe shrinks, as shown in Figure 15a. The operation of the device can be simplified to a double-hinged semi-circular arch model, as shown in Figure 15b. When the hole-wall rock compresses the Q-CFSD until the pipe wall completely wraps the cable bolt, the relative displacement of the semicircular arch model bearing is expressed as follows:

(3) $$\Delta l=2(R_{0}-r_{0})$$

where Δl is the relative displacement of the semicircular arch model bearing; *R*_0_ is the average radius of the slotted pipe; and *r*_0_ is the cable-bolt radius. Ignoring friction between the pipe walls on the left side of the model, according to static calculation results for a double-hinged arch structure [43], the shear resistance provided by the steel pipe is expressed as follows:

(4) F1=15EpIpL16R03(R0−r0−t2)

where *F*_1_ is the shear resistance provided by the CFSD at the shrinkage deformation stage, and *L* is the length of the CFSD; and *E*_p_ and *I*_p_ are Young’s modulus and the inertial moment of the slotted pipe section, respectively.

Once the slotted steel pipe is completely wrapped around the cable bolt, the pipe wall is continuously tightened as the shear displacement increases, forming a uniform radial pressure around the cable bolt, as shown in Figure 14c. The concentrated shear forces from the top and bottom of the cable bolt are eventually transformed into uniform radial pressure, and the shear stress concentration is thus reduced. The slotted steel pipe and cable bolt work together and are considered as a pipe–cable bearing whole. Therefore, their effect is equivalent to a solid circular beam model, as shown in Figure 15d. In the range of joint-plane shear action, both a bending moment and shear force exist in the pipe–cable bearing whole section; therefore, deformation of this beam occurs as transverse bending. According to material mechanics, the maximum shear stress on the pipe–cable bearing whole cross-section on the joint plane (Figure 15d) is expressed as follows [44]:

(5) $$\tau_{\max}=\frac{F_{2}S_{z}^{*}}{I_{z}d}$$

where $\tau_{\max}$ is the maximum shear stress on the pipe–cable bearing whole section; *F*_2_ is the shear resistance provided by the pipe–cable bearing whole; *d* is the diameter of the pipe–cable bearing whole cross-section along the neutral axis *z*; *I*_z_ denotes the moment of inertia of the pipe–cable bearing whole cross-section applied to the neutral axis *z*, $I_{z}=\int y^{2}dA=\frac{\pi d^{4}}{64}$; $S_{z}^{*}$ denotes the static moment of the pipe–cable bearing whole cross-section applied to the neutral axis *z*, $S_{z}^{*}=\int ydA=\frac{d^{3}}{12}$; and *A* is the cross-sectional area of the pipe–cable bearing whole. Assuming that the shear strength of the Q-CFSD is lower than that of the cable bolt, the steel pipe will break before the cable bolt. Setting Equation (5) to be equal to the shear strength of the steel-pipe section, the shear resistance provided by the pipe–cable bearing whole can then be obtained as follows:

(6) $$F_{2}=\frac{3}{4}\tau_{f2}A$$

From Equations (4) and (6), the shear resistance provided by the cable bolt with the CFSDs is positively correlated with the shear strength, cross-sectional area, and elastic modulus of the CFSD. Thus, it is confirmed that the shear resistance of the anchor cable system to the surrounding rock can greatly improve by adding CFSDs, which, to a certain extent, alleviates the contradiction between the high axial tension and low shear resistance of the anchor cable system.

## 7. Conclusions

To address the problem of relatively weak shear resistance and easily damaged free sections of end-anchored cable bolts used in coal-mine roadways, under the shearing action of surrounding rock, this study proposes a strengthening scheme wherein high-strength steel pipes are used to protect the sheared cable bolts. Design of a strengthening device is presented. Through numerical simulation, theoretical analysis, and laboratory tests, this study determined the optimal device type, elucidated its strengthening mechanism, and verified its operation. The main conclusions are as follows:

Cable-bolt failure is caused by the combined action of axial tension and transverse shear under the shearing action of surrounding rock mass. The shear force that the cable bolt can bear is negatively correlated with its axial force. Additionally, surface shear damage and uneven distribution of shear stress on the cable-bolt section exacerbate the occurrence of tension–shear failure.

Avoiding surface shear damage and section stress concentration and improving shear resistance of the cable bolt were set as the main objectives of this study. A basic CFSD structure was designed. Numerical simulation results revealed that the CFSD could effectively strengthen the shear resistance and deformation resistance of the cable bolt and better promote axial load transfer. Additionally, the CFSD can also protect the cable bolt from harmful plastic damage and improve shear stress concentration. The Q-CFSD that incorporated a slotted steel pipe achieved the best comprehensive performance and was selected as the device prototype.

The results obtained by theoretical analysis revealed that the shear resistance provided by the reinforced cable bolt positively correlated with the shear strength, cross-sectional area, and elastic modulus of the CFSD. The shear resistance of the cable-support system to the surrounding rock can be improved by adding more CFSDs. The laboratory test results revealed that, compared with the findings for a normal cable bolt, the peak shear force on the joint plane, the peak cable axial force, and the ultimate shear displacement increased by 31%, 18%, and 11%, respectively, in a double-shear experiment on the cable bolt with CFSDs. The CFSD was highly capable of adapting to the cable-bolt deformation and formed a shear whole with the bolt, which not only increased its shear resistance and deformation resistance, but also improved axial tensile performance.

## Figures and Tables

**Table 1:** Design parameters of the strengthening device.

Wall Thickness (mm)	Outer Diameter (mm)	Density (kg/m^3^)	Yield Strength (MPa)	Peak Strength (MPa)	Elongation (%)
2	25	7800	355	600	16

**Table 2:** Model loading analysis steps.

Analysis Step	Tensile Displacement *U*_0_ (mm)	Confining Pressure *p*_0_ (MPa)	Shear Speed *V*_1_ (mm/s)	Step Time *T* (s)	Diagram of Loading Process
Step-1	1.7	1	0	1	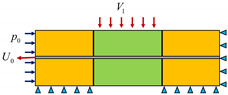
Step-2	0	0	0.2	250	

**Table 3:** Input mechanical properties of rock and steel-pipe models.

Material Type	Density (kg/m^3^)	Poisson’s Ratio	Young’s Modulus (GPa)	Friction Angle (°)	Cohesion (MPa)	Yield Strength (MPa)	Peak Strength (MPa)	Peak Strain (%)
Rock	2600	0.2	21.0	30.0	4.1	—	—	—
Steel pipe	7800	0.3	206	—	—	355	600	10

**Table 4:** Input material property parameters of cable bolt.

Density (kg/m^3^)	Poisson’s Ratio	Young’s Modulus (GPa)	Ultimate Elastic Strain	Tensile Fracture Strain	Shear Fracture Strain	Displacement at Failure (mm)
7800	0.3	210	0.008	0.06	0.02	0.3

**Table 5:** Results of strength parameters and increments of numerical simulations.

Schemes	Peak Shear Force (kN)	Increment (%)	Maximum Axial Force (kN)	Increment (%)	Ultimate Shear Displacement (mm)	Cable Shear Displacement (mm)
S1-N	283	-	258	-	34.7	25
S2-O	397	40.3	314	21.7	40.4	34
S3-C	396	39.9	304	17.8	38.4	32
S4-Q	399	41.0	312	20.9	40.3	34

## Data Availability

The data used to support the findings of this study are available from the corresponding author upon request.
